# Novel retrograde puncture method to establish preperitoneal space for laparoscopic direct inguinal hernia repair with internal ring suturing

**DOI:** 10.1590/1414-431X20165247

**Published:** 2016-05-13

**Authors:** H. Jiang, R. Ma, X. Zhang

**Affiliations:** Department of General Surgery, General Hospital of Shenyang Military Area Command, Shenyang, Liaoning, China

**Keywords:** Laparoscopy, Retrograde puncture, Direct inguinal hernia, Inguinal ring suture

## Abstract

The aim of this study was to explore the clinical efficacy of a novel retrograde puncture approach to establish a preperitoneal space for laparoscopic direct inguinal hernia repair with inguinal ring suturing. Forty-two patients who underwent laparoscopic inguinal hernia repair with retrograde puncture for preperitoneal space establishment as well as inguinal ring suturing between August 2013 and March 2014 at our hospital were enrolled. Preperitoneal space was successfully established in all patients, with a mean establishment time of 6 min. Laparoscopic repairs were successful in all patients, with a mean surgical time of 26±15.1 min. Mean postoperative hospitalization duration was 3.0±0.7 days. Two patients suffered from postoperative local hematomas, which were relieved after puncturing and drainage. Four patients had short-term local pain. There were no cases of chronic pain. Patients were followed up for 6 months to 1 year, and no recurrence was observed. Our results demonstrate that preperitoneal space established by the retrograde puncture technique can be successfully used in adult laparoscopic hernioplasty to avoid intraoperative mesh fixation, and thus reduce medical costs.

## Introduction

Inguinal hernia is a common disease (1) for which surgical treatment is the most effective therapeutic approach (2). Laparoscopic hernia repair involves the clinical application of minimally invasive technique (3 – 5). Totally extraperitoneal hernia repair (TEP) and transabdominal preperitoneal repair (TAPP) are the main surgical approaches for laparoscopic hernia repair (6–8). Totally extraperitoneal hernia repair for patients with hernia of the inner ring has the advantage of being minimally traumatic, safe, having few complications and low recurrence rate (9 – 11).

During TEP, establishment of preperitoneal space using different methods is the key to a successful surgery (12), including the percutaneous balloon separation method, the suprapubic puncture method, the finger separation method, and the direct mirror push method (13). McKernan et al. (2) were the first to use the percutaneous balloon separator establishing the preperitoneal space. This method requires a special casing and has a high-cost. Suprapubic puncture method can easily cause bowel injury during the blind insertion of the catheter, increasing the risk of infection (14). The finger separation method, which produces a large incision and has a high risk of injury to the peritoneum, is currently the most common technique (15). The direct mirror push method needs the lens to be wiped and the abdominal wall is slowly separated without the help of other instruments (16). Based on the above-described disadvantages of the method, in this study we aimed to introduce a novel approach for the establishment of preperitoneal space during TEP, using the retrograde puncture technique.

In 2004, Moreno-Egea et al. (17) found that the recurrence rate of direct hernia in patients undergoing TEP without fixation was relatively high. In 2012, the guidelines of the International Endohernia Society (IEHS) (18) indicated that tacker fixation could increase the incidence of acute and chronic pain as well as medical costs, and recommended fixation for type III hernias (particularly for direct hernias), but not for type I and II hernias. In our TEP surgeries, we also adopted the internal ring suturing technique to avoid fixations and mesh repairs for patients with bilateral direct hernias or hernias >3 cm, and achieved excellent results.

## Material and Methods

The study protocol was approved by the Ethics Committee of the General Hospital of Shenyang Military Area Command, and all participants provided written informed consent.

Of the 42 patients, 19 were males and 23 females. The mean age was 50.4±13.4 years. Fifteen patients had an internal ring diameter >3 cm, while the remaining 27 had a diameter <3 cm. There were 32 cases of unilateral direct hernias (including those with a contralateral oblique hernia), and 10 cases of bilateral direct hernias (19).

## Surgical techniques

**Figure 1:**
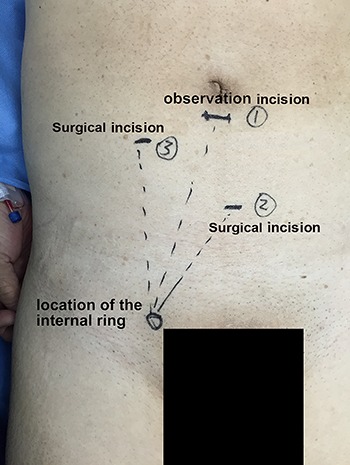
Location of the incisions.

**Figure 2:**
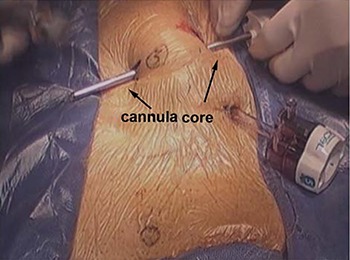
Puncturing of the 5 mm cannula core out of the skin from the superficial surface of the posterior rectus sheath.

**Figure 3:**
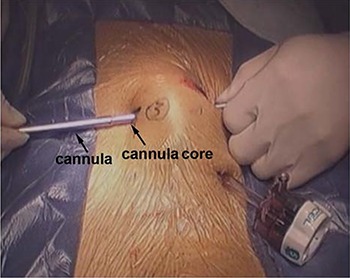
Delivering the cannula into the preperitoneal space under the guidance of the cannula core.

**Figure 4:**
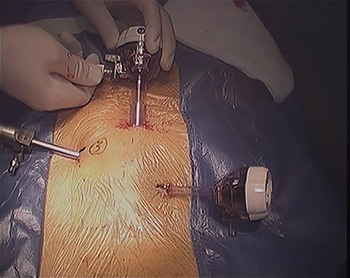
Surgical field after all 3 cannulas were inserted successfully.

**Figure 5:**
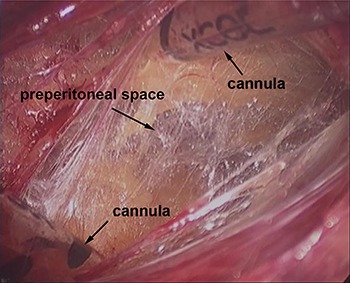
Separating the preperitoneal space with the two surgery cannulas.

In TEP, general anesthesia guided by endotracheal intubation or laryngeal mask, and preoperative indwelling urinary catheterization were applied to the patients who were in a horizontal position. Surgeons were standing contralaterally to the surgical site and the monitor was placed over the surgical site. A triangular puncture, with the two surgical incisions located bilaterally to the observation incision, was made (Figure 1). Preperitoneal space was established using retrograde puncture, which created a 1 cm transverse incision at the hernia site 1 cm below the umbilicus, penetrated through the skin and subcutaneous tissues, and opened the anterior rectus sheath. Subsequently, the rectus abdominis muscle was pulled open with a wire retractor, and the space created between the rectus abdominis muscle and posterior rectus sheath was filled with gauze. The cannula core (Ethion Endo-surgery, LLC.475 Calle C, USA) was inserted through the incision to puncture through the skin at the site preserved for the surgical incision, between the posterior rectus sheath and the rectus abdominis muscle. The cannula was then inserted into the preperitoneal space along the core (Figures 2 and 3). The other surgery cannula was inserted into the preperitoneal space laterally to the rectus abdominis in the same manner. Finally, the 12-mm cannula was also inserted into the preperitoneal space through the incision. After the successful establishment of the three cannulas, regular inflation was performed, and the endoscope was inserted. After endoscopically locating the two surgery cannulas, they were used to bluntly dissect the muscles and complete the establishment of the preperitoneal space (Figures 4 and 5). If the space was adequate, an ultrasonic scalpel and atraumatic grasping forceps were inserted to further expand the preperitoneal space. Since the sac of the direct hernia can be easily retracted into the preperitoneum, the internal ring was sutured after separating the hernia.

**Figure 6:**
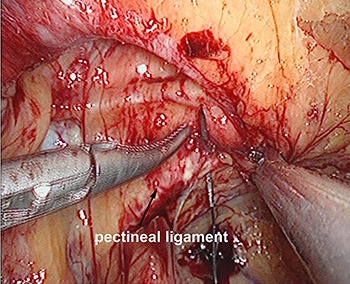
Pectineal ligament was punctured using the suture needle.

During internal ring suturing, we had a clear vision of the internal ring after establishing the preperitoneal space and separating the hernia. After measuring the internal ring diameter, we performed the internal ring suturing using the following methods: the pectineal ligament was punctured using the suture needle, and the false sac was pulled into the preperitoneal space; the suture needle was then used to penetrate the base of the sac and puncture through the conjoined tendon arch; as a result, the upper and lower edges of the defect as well as the false sac were sutured together after knotting (Figures 6–9). Notably, the aforementioned sutures were not tightly knotted, since our primary goal was to shrink the hernia ring and add intervals inside the ring to increase its resistance to the mesh plug. The #0 absorbable sutures were used for suturing.

**Figure 7 f07:**
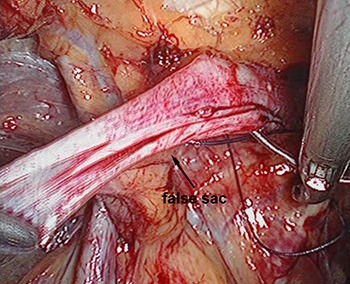
Suture needle was used to penetrate the base of the false sac.

**Figure 8 f08:**
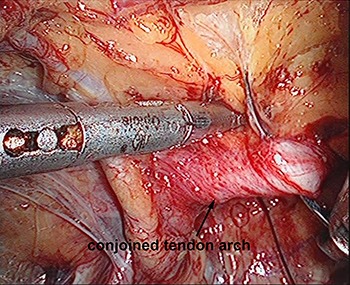
Puncture through the conjoined tendon arch.

**Figure 9:**
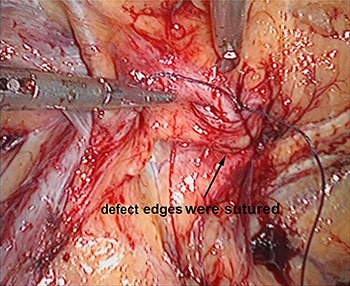
Upper and lower edges of the defect were sutured together.

For the mesh plug placement, the Bard 3DMax mesh (Davol Inc., USA) was placed and flattened at the unfixed myopectineal orifice. The mesh was medially placed across the midline to laterally cover the internal ring and spermatic cord (in male patients), extend into the iliopsoas fascia, inferiorly cross the pectineal ligament, and enter the space of Retzius. Slow inflation with CO_2_ was performed to fix the mesh at its designated site.

## Results

Preperitoneal space establishment was successfully performed in all cases, and no bleeding complications occurred during the process. The mean establishment time for the preperitoneal space was 6 min. Laparoscopic hernia repair was successful in all patients, with a mean surgery time of 26±15.1 min and mean hospitalization duration of 3.0±0.7 days. Two patients at the early stage of the study suffered from postoperative local hematomas due to false sac effusion caused by incomplete suturing between the false sac base and hernia ring, which was relieved after puncturing and drainage. Four patients had short-term local pain. There were no cases of chronic pain. Patients were followed up for 6 months to 1 year, and no recurrence was observed.

## Discussion

Establishment of a preperitoneal space is the key step in TEP. We have created the retrograde puncture method, which can completely eliminate damage to the peritoneum by establishing the preperitoneal space, with the cannula core puncturing through the preperitoneal space from inside, and guiding the cannula to the preperitoneal space. Before inflation and endoscopic dissection of the preperitoneal space, the three cannulas required in the surgery are already in position, which reduces the surgery time and simplifies the procedure. After inflation and endoscope placement, further dissection of the preperitoneal space can be easily achieved using the two aforementioned puncture cannulas, preventing image blurring during dissection with endoscopy.

Whether a mesh plug is needed in the laparoscopic inguinal hernia repair (LIHR) remains controversial (20 – 22). Earlier, mesh fixations were performed using a fibrin sealant or sutures (23), which led to complications such as foreign body sensation, paresthesia, and acute and chronic pain, and thus increased the medical costs (24–27). In 1995, Dunn (28) questioned the necessity of mesh plug placement. In 2004, Moreno-Egea et al. (17) found a high recurrence rate in patients undergoing TEP without mesh fixation. In 2006, Koch et al. (29) claimed that mesh fixation was not needed for defects with a diameter <3 cm. In 2008, Taylor et al. (21) reported that patients with a defect <2 cm could safely undergo a repair surgery without mesh fixation. According to IEHS guidelines (18), mesh fixation is required in TEP for direct hernia. In our practice of internal ring suturing, we noticed that the internal ring is already occluded before mesh fixation, and recurrence does not seem to occur in the short term even if mesh fixation was not performed (30). However, better results might be achieved if the repair is reinforced with a mesh fixation.

During LIHR for direct hernia, the false sac base should be sutured together with the internal ring, or local hematoma will occur since effusion of the false sac cannot be drained after the internal ring is closed. In the first two surgeries, we did not suture the false sac base with the internal ring, and effusion occurred due to insufficient drainage. After drainage, the symptoms were relieved.

The surrounding structures of the hernia ring are simple (31), with the pectineal ligament located below and the conjoined tendon arch above. Thus, suturing of the hernia ring is convenient and easy. However, structures seen under the endoscopic surgery field are displayed in two dimensions (32), so suturing could be difficult. The suturing site of the hernia ring is similar to "suturing on the ceiling", which increases the surgical difficulty. Moreover, femoral, inferior epigastric, and obturator vessels penetrate through the posterior wall of the myopectineal orifice (33), and inaccurate surgery will result in damage (34). Therefore, this surgery should be performed only by surgeons who are highly skilled in endoscopic surgery and suturing (35). In our opinion, the endoscopic suturing and knotting is a convenient technique to master with regular practicing, which should greatly reduce medical costs. Before performing the LIHR surgeries in this study, we had extensively practiced with a simulator and skillfully mastered the techniques of suturing and knotting. Therefore, complications associated with internal ring suturing did not occur in our study.

In conclusion, our results demonstrate the successful application of retrograde puncture in establishing the preperitoneal space during an LIHR. LIHR with hernia ring suturing can prevent mesh fixation in adult patients with inguinal hernias, thereby avoiding complications such as pain and bleeding, and also lowering the medical costs. In addition, this technique may broaden the indications for laparoscopic herniorrhaphy to also include patients with large internal ring defects. However, difficult suturing is a limitation of the retrograde puncture technique. We have not yet assessed this novel approach in a large-scale clinical report but the pros and cons of this approach will be further clarified as more surgical cases are carried out.

## References

[1] Moreno-Egea A, Flores B, Girela E, Martin JG, Aguayo JL, Canteras M (2002). Spigelian hernia: bibliographical study and presentation of a series
of 28 patients. Hernia.

[2] McKernan JB, Laws HL (1993). Laparoscopic repair of inguinal hernias using a totally
extraperitoneal prosthetic approach. Surg Endosc.

[3] Alvarez C (2004). Open mesh versus laparoscopic mesh hernia repair. N Engl J Med.

[4] Lowham AS, Filipi CJ, Fitzgibbons RJ, Stoppa R, Wantz GE, Felix EL (1997). Mechanisms of hernia recurrence after preperitoneal mesh repair.
Traditional and laparoscopic. Ann Surg.

[5]  Parker M, Bray JM, Pfluke JM, Asbun HJ, Smith CD, Bowers SP (2011). Preliminary experience and development of an algorithm for the optimal
use of the laparoscopic component separation technique for myofascial advancement
during ventral incisional hernia repair. J Laparoendosc Adv Surg Tech A.

[6] Bracale U, Melillo P, Pignata G, Di Salvo E, Rovani M, Merola G (2012). Which is the best laparoscopic approach for inguinal hernia repair:
TEP or TAPP? A systematic review of the literature with a network
meta-analysis. Surg Endosc.

[B07] Antoniou SA, Antoniou GA, Bartsch DK, Fendrich V, Koch OO, Pointner R (2013). Transabdominal preperitoneal versus totally extraperitoneal repair of
inguinal hernia: a meta-analysis of randomized studies. Am J Surg.

[8] Schrenk DP, Woisetschläger R, Rieger R, Wayand W (1996). Prospective randomized trial comparing postoperative pain and return
to physical activity after transabdominal preperitoneal, total preperitoneal or
Shouldice technique for inguinal hernia repair. Br J Surg.

[9] Horisberger K, Jung MK, Zingg U, Schob O (2013). Influence of type of mesh fixation in endoscopic totally
extraperitoneal hernia repair (TEP) on long-term quality of life. World J Surg.

[10] McCormack K, Wake BL, Fraser C, Vale L, Perez J, Grant A (2005). Transabdominal pre-peritoneal (TAPP) versus totally extraperitoneal
(TEP) laparoscopic techniques for inguinal hernia repair: a systematic
review. Hernia.

[11] Misra MC, Kumar S, Bansal VK (2008). Total extraperitoneal (TEP) mesh repair of inguinal hernia in the
developing world: comparison of low-cost indigenous balloon dissection versus
direct telescopic dissection: a prospective randomized controlled
study. Surg Endosc.

[12] Beets GL, Dirksen CD, Go PM, Geisler FE, Baeten CG, Kootstra G (1999). Open or laparoscopic preperitoneal mesh repair for recurrent inguinal
hernia? A randomized controlled trial. Surg Endosc.

[13] Li JW (2010). [The main technical points of laparoscopic inguinal hernia
repair]. J Lapar Surg.

[14] Tetik C, Arregui ME, Dulucq JL, Fitzgibbons RJ, Franklin ME, McKernan JB (1994). Complications and recurrences associated with laparoscopic repair of
groin hernias. A multi-institutional retrospective analysis. Surg Endosc.

[15] Bringman S, Ek A, Haglind E, Heikkinen TJ, Kald A, Kylberg F (2001). Is a dissection balloon beneficial in bilateral, totally
extraperitoneal, endoscopic hernioplasty? A randomized, prospective, multicenter
study. Surg Laparosc Endosc Percutan Tech.

[16] Li JW (2009). [Advertent questions in laparoscopic hernioplasty]. Hernia.

[17] Moreno-Egea A, Torralba Martinez JA, Morales Cuenca G, Aguayo Albasini JL (2004). Randomized clinical trial of fixation vs nonfixation of mesh in total
extraperitoneal inguinal hernioplasty. Arch Surg.

[18] Berney CR (2012). Guidelines for laparoscopic (TAPP) and endoscopic (TEP) treatment of
inguinal hernia. Surg Endosc.

[19] Muysoms FE, Miserez M, Berrevoet F, Campanelli G, Champault GG, Chelala E (2009). Classification of primary and incisional abdominal wall
hernias. Hernia.

[20] Bell RC, Price JG (2003). Laparoscopic inguinal hernia repair using an anatomically contoured
three-dimensional mesh. Surg Endosc.

[21] Taylor C, Layani L, Liew V, Ghusn M, Crampton N, White S (2008). Laparoscopic inguinal hernia repair without mesh fixation, early
results of a large randomised clinical trial. Surg Endosc.

[22] Sajid MS, Ladwa N, Kalra L, McFall M, Baig MK, Sains P (2013). A meta-analysis examining the use of tacker mesh fixation versus glue
mesh fixation in laparoscopic inguinal hernia repair. Am J Surg.

[23] Moreno-Egea A, Castillo JA, Girela E, Canteras M, Aguayo JL (2002). Outpatient laparoscopic incisional/ventral hernioplasty: our
experience in 55 cases. Surg Laparosc Endosc Percutan Tech.

[24] Anthony T, Bergen PC, Kim LT, Henderson M, Fahey T, Rege RV (2000). Factors affecting recurrence following incisional
herniorrhaphy. World J Surg.

[B25] Mudge M, Hughes LE (1985). Incisional hernia: a 10 year prospective study of incidence and
attitudes. Br J Surg.

[B26] Millikan KW (2003). Incisional hernia repair. Surg Clin North Am.

[27] Bencini L, Sanchez LJ, Boffi B, Farsi M, Scatizzi M, Moretti R (2003). Incisional hernia: repair retrospective comparison of laparoscopic and
open techniques. Surg Endosc.

[28] Dunn DC (1995). Laparoscopic hernia repair without the use of staples or knotting
manoeuvres. Br J Surg.

[29] Koch CA, Greenlee SM, Larson DR, Harrington JR, Farley DR (2006). Randomized prospective study of totally extraperitoneal inguinal
hernia repair: fixation versus no fixation of mesh. JSLS.

[30] Tran H (2011). Safety and efficacy of single incision laparoscopic surgery for total
extraperitoneal inguinal hernia repair. JSLS.

[31] Klinge U, Conze J, Limberg W, Brucker C, Ottinger AP, Schumpelick V (1996). [Pathophysiology of the abdominal wall]. Chirurg.

[32] Sadava EE, Schlottmann F, Bun ME, Rotholtz NA (2016). Laparoscopic incisional hernia repair after colorectal surgery. Is it
possible to maintain a mini-invasive approach?. Surg Endosc.

[33] Hua W, Liang ZH, Zhao XW, Ding JW, Tang ZP, Ding ZH (2014). [The clinical use and significance of the spatial anatomical
separation of myopectineal orifice in totally extraperitoneal prosthesis for
repair of inguinal hernia]. Chin J Clin Anat.

[34] Toy FK, Bailey RW, Carey S, Chappuis CW, Gagner M, Josephs LG (1998). Prospective, multicenter study of laparoscopic ventral hernioplasty.
Preliminary results. Surg Endosc.

[35] Stylopoulos N, Gazelle GS, Rattner DW (2003). A cost-utility analysis of treatment options for inguinal hernia in
1,513,008 adult patients. Surg Endosc.

